# Can differences in phosphorus uptake kinetics explain the distribution of cattail and sawgrass in the Florida Everglades?

**DOI:** 10.1186/1471-2229-10-23

**Published:** 2010-02-08

**Authors:** Hans Brix, Bent Lorenzen, Irving A Mendelssohn, Karen L McKee, ShiLi Miao

**Affiliations:** 1 Department of Biological Sciences, Aarhus University, Ole Worms Allé 1, DK-8000 Århus C, Denmark; 2 Department of Oceanography and Coastal Sciences, Louisiana State University, Baton Rouge, LA 70803, USA; 3 US Geological Survey, National Wetlands Research Center, Lafayette, LA 70506, USA; 4 South Florida Water Management District, West Palm Beach, FL 33406, USA

## Abstract

**Background:**

Cattail (*Typha domingensis*) has been spreading in phosphorus (P) enriched areas of the oligotrophic Florida Everglades at the expense of sawgrass (*Cladium mariscus *spp. *jamaicense*). Abundant evidence in the literature explains how the opportunistic features of *Typha *might lead to a complete dominance in P-enriched areas. Less clear is how *Typha *can grow and acquire P at extremely low P levels, which prevail in the unimpacted areas of the Everglades.

**Results:**

Apparent P uptake kinetics were measured for intact plants of *Cladium *and *Typha *acclimated to low and high P at two levels of oxygen in hydroponic culture. The saturated rate of P uptake was higher in *Typha *than in *Cladium *and higher in low-P acclimated plants than in high-P acclimated plants. The affinity for P uptake was two-fold higher in *Typha *than in *Cladium*, and two- to three-fold higher for low-P acclimated plants compared to high-P acclimated plants. As *Cladium *had a greater proportion of its biomass allocated to roots, the overall uptake capacity of the two species at high P did not differ. At low P availability, *Typha *increased biomass allocation to roots more than *Cladium*. Both species also adjusted their P uptake kinetics, but *Typha *more so than *Cladium*. The adjustment of the P uptake system and increased biomass allocation to roots resulted in a five-fold higher uptake per plant for *Cladium *and a ten-fold higher uptake for *Typha*.

**Conclusions:**

Both *Cladium *and *Typha *adjust P uptake kinetics in relation to plant demand when P availability is high. When P concentrations are low, however, *Typha *adjusts P uptake kinetics and also increases allocation to roots more so than *Cladium*, thereby improving both efficiency and capacity of P uptake. *Cladium *has less need to adjust P uptake kinetics because it is already efficient at acquiring P from peat soils (e.g., through secretion of phosphatases, symbiosis with arbuscular mycorrhizal fungi, nutrient conservation growth traits). Thus, although *Cladium *and *Typha *have qualitatively similar strategies to improve P-uptake efficiency and capacity under low P-conditions, *Typha *shows a quantitatively greater response, possibly due to a lesser expression of these mechanisms than *Cladium*. This difference between the two species helps to explain why an opportunistic species such as *Typha *is able to grow side by side with *Cladium *in the P-deficient Everglades.

## Background

The wetland species, *Cladium mariscus *ssp. *jamaicense *(L.) Pohl (Crantz) K. Kenth (sawgrass; hereafter *Cladium*) and *Typha domingensis *Pers. (cattail; hereafter *Typha*) are both native to the Florida Everglades and occupy similar habitats [1]. *Cladium *was the dominant plant species in the historical freshwater Everglades, whereas *Typha *was a minor species occurring in small and scattered patches throughout the Everglades [2]. However, during the past decades *Typha *has expanded rapidly and replaced thousands of hectares of *Cladium *marshes and aquatic slough areas in the northern part of the Everglades [3-6]. Numerous studies have been conducted to assess the causes and the consequences of this change in vegetation and community structure [7-20], and the driving force for the change appears to be nutrient enrichment, particularly phosphorus (P), from agricultural runoff and Lake Okeechobee outflow [21].

*Cladium *and *Typha *are both large, clonal species that can form monospecific communities in freshwater habitats. The two species differ, however, in morphology, growth, and life history characteristics [10,15,22]. *Cladium *exhibits many characteristics of adaptation to infertile environments, such as slow growth rate, long leaf longevity, low capacity for nutrient uptake, low leaf nutrient concentrations and a relatively inflexible partitioning of biomass in response to increased nutrient availability [23,24]. *Typha*, on the other hand, has traits of an opportunistic species from nutrient-rich habitats with high growth rates, short leaf longevity, high capacity for nutrient uptake, high leaf nutrient concentrations and flexible biomass partitioning [8,25]. Both species are adapted to grow in waterlogged soils by virtue of a well-developed aerenchyma system, but convective gas flow has been documented only in *Typha *and not in *Cladium *[26-29]. Furthermore, *Cladium *has lower root porosity and generally higher alcoholic fermentation rates, indicating lower capacity for root aeration than *Typha *[30]. These inherently different traits are considered the main explanation for the rapid spread and competitive success of *Typha *in the P-enriched areas of the Florida Everglades.

*Cladium *and *Typha *also co-exist in the oligotrophic areas of the Florida Everglades where P availability is extremely low. In the interior of Water Conservation Area 2A, an impounded area in the northern Everglades, *Cladium *and *Typha *grow together despite soluble P concentrations of less than 4 μg l^-1 ^in the porewaters throughout the soil profile [31]. *Typha *is much less abundant than *Cladium *and has slow growth rates in these areas [32], and although nutrient enrichment and disturbance around alligator holes have been suggested to favour the proliferation of *Typha *locally [33], the traits that allow the growth of a high resource-adapted plant like *Typha *in this low P environment are not understood.

Studies at high P availability have demonstrated that *Typha *has a greater relative growth rate, a greater allocation of biomass to leaves, and a lower P-use efficiency than *Cladium *[10,15,16]. In fertile habitats, a high nutrient uptake capacity per unit of root biomass and a high growth rate and biomass allocation to leaves increase the capability to compete for light and reduce the need for a high root biomass. However, these traits are not advantageous for growth in a nutrient deficient environment where plants must acquire nutrients at low availability and minimize nutrient losses [34]. In such conditions, optimal features would include an extensive root system for soil exploration, a high root surface area (long, thin roots and/or root hairs) for acquisition of nutrients, and efficient mechanisms to capture nutrient ions at low external concentrations [35-37].

The main research question we address here is: Which characteristics of *Cladium *and *Typha *allow the species to grow in the oligotrophic P-deficient interior of the Florida Everglades, and at the same time explain why *Typha *out-competes *Cladium *under P-enriched conditions? As to the second part of the question, abundant evidence in the literature explains how the opportunistic features of *Typha *can lead to complete dominance in P-enriched areas [e.g. [7,8,13,15]]. Less clear is how *Typha *can grow and acquire P at the extreme low P-levels prevailing in the unimpacted areas of the Everglades.

We hypothesized that *Typha *has a more plastic P uptake system than *Cladium *in relation to P availability, and this strategy will allow adequate uptake of P and better competitive ability over a wide range of external P concentrations. Furthermore, we hypothesized that oxygen-deficient conditions will affect the uptake kinetics of *Cladium *more than that of *Typha*, as the latter species has a more efficient system for root aeration (via aerenchyma and internal convective gas flow). These hypotheses were tested in a series of P uptake experiments designed to distinguish differences in P uptake kinetics between the two species.

Apparent P uptake kinetics were measured for whole plants of *Cladium *and *Typha *grown from seeds and acclimated to identical, steady state conditions, in a factorial treatment arrangement with two levels of P (5 and 500 μg P l^-1^) and two levels of oxygen (8.0 and <0.5 mg O_2 _l^-1^) in hydroponic culture solutions (n = 4-8). The P uptake kinetic parameters were estimated using a modified Michaelis-Menten model [38-41].

## Results

### Plant characteristics

The plants used in the uptake studies all appeared healthy, with no visual signs of nutrient deficiencies. However, long acclimation periods in the various culture combinations (up to 4 months in the low P treatments) inevitably created plants with different biomass, allocation patterns, and tissue nutrient concentrations. Overall the shoot height (average 0.95 m) and root length (average 0.37 m) of the two species varied little across the treatments, but the plant weights and biomass allocation differed significantly (table 1). Low-P acclimated plants had less biomass than high-P acclimated plants, and significantly (P < 0.001) more biomass allocated to roots. *Typha *in particular allocated less biomass to roots in the high P treatment (table 1). Plants in the low oxygen cultures had 25% shorter root systems than those in aerated cultures (P < 0.001), but the oxygen treatment did not affect the proportion of biomass allocated to roots (P > 0.05).

**Table 1:** Plant size

Species	Oxygen	P	Leaf length (m)	Root length (m)	Plant weight (g DW)	Root fraction (%)
*Cladium*	High oxygen	Low	1.01 ± 0.05	0.30 ± 0.03	5.6 ± 0.7	17.4 ± 1.9
		High	0.98 ± 0.09	0.50 ± 0.10	7.0 ± 1.9	16.1 ± 1.6
	Low oxygen	Low	1.00 ± 0.12	0.32 ± 0.04	7.0 ± 1.9	18.3 ± 2.2
		High	0.95 ± 0.08	0.30 ± 0.06	12.8 ± 2.9	14.1 ± 2.2

*Typha*	High oxygen	Low	0.69 ± 0.12	0.48 ± 0.05	5.8 ± 0.5	22.3 ± 1.9
		High	0.96 ± 0.05	0.42 ± 0.06	11.6 ± 1.4	8.7 ± 1.6
	Low oxygen	Low	0.96 ± 0.06	0.39 ± 0.03	9.1 ± 1.3	15.1 ± 2.0
		High	1.08 ± 0.09	0.29 ± 0.04	23.6 ± 4.3	7.7 ± 0.6

Average (± SE, n = 4-8) leaf and root length, total plant weight and percentage of biomass allocated to roots of the *Cladium mariscus *spp. *jamaicense *and *Typha domingensis *plants acclimated to a low and a high P level (5 and 500 μg P l^-1^) and to aerated and low oxygen conditions (8.0 and <0.5 mg O_2 _l^-1^) in hydroponic culture solutions

**Figure 1:**
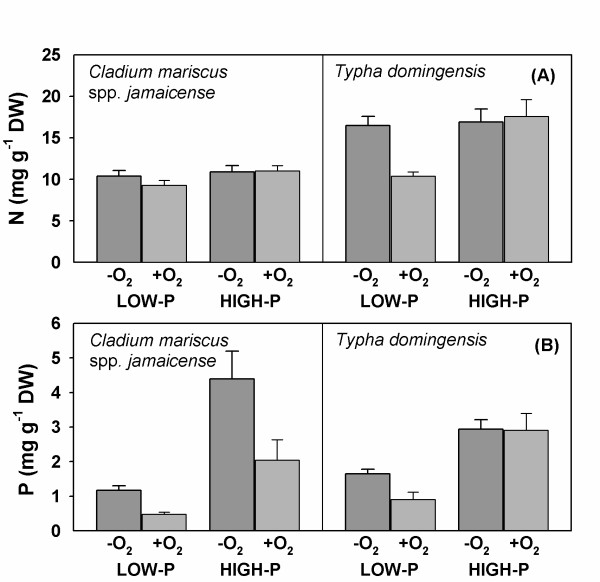
**Plant tissue N and P concentrations**. Average (± SE) concentrations of nitrogen (N) and phosphorus (P) (mg g^-1 ^dry weight) in whole plants of *Cladium mariscus *spp. *jamaicense *and *Typha domingensis *acclimated to low (5 μg l^-1^) and high (500 μg l^-1^) P concentrations and high (+O_2_: 8 mg l^-1^) and low (-O_2_: <0.5 mg l^-1^) oxygen concentration in the culture solutions.

The tissue N concentrations in *Cladium *were similar (average 10.4 mg g^-1 ^dry weight) in all treatments, and lower overall than in *Typha *(approximately 17 mg g^-1 ^dry weight), except in the aerated, low-P treatment where plants were smaller (figure 1). The tissue P concentrations were, as expected, more variable across treatments, and were generally higher (P < 0.001) in the high P treatment (average 3.1 mg g^-1 ^dry weight) than in the low P treatment (average 1.1 mg g^-1 ^dry weight). Moreover, the tissue P concentrations were consistently higher in the low oxygen treatments than in the corresponding aerated treatments (figure 1). However, tissue P analyses were carried out on tissues harvested after the uptake kinetic studies, in which plants were exposed to high P concentrations (up to 500 μg l^-1^) for several hours. Uptake during the uptake studies may have contributed to enhance the tissue concentration by 0.4-0.7 mg P g^-1 ^dry weight. Hence, the tissue P concentrations in the low-P acclimated plants presented here are likely higher than they would have been if plants were collected for analysis before the uptake kinetic studies.

### Phosphorus uptake kinetics

**Figure 2:**
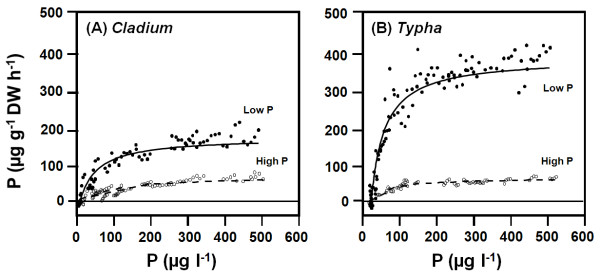
**P uptake kinetic curves**. Examples of relationships between the concentration of inorganic phosphorus (P) in the root chambers and the rate of P uptake of single specimens of (A) *Cladium mariscus *spp. *jamaicense *and (B) *Typha domingensis *acclimated to low (5 μg l^-1^) P concentrations (solid lines and closed symbols) and to high (500 μg l^-1^) P concentrations (dashed lines and open symbols). Plants were grown at low (<0.5 mg l^-1^) oxygen concentration in culture solutions. Uptake was measured by P depletions of root solutions followed by stepwise increments of the P concentrations. The relationship was fitted to a modified Michaelis-Menten model (curves).

The relationships between solution P concentrations and the net rate of P uptake of selected *Cladium *and *Typha *plants acclimated to low P and to high P levels are shown in figure 2. Generally, the modified Michaelis-Menten model fitted the relationships and estimated the kinetic uptake parameters with high statistical confidence. V_max _was entered as a fixed parameter in the model and was estimated as the average uptake rate at solution P concentrations where uptake appeared to be saturated. At the low concentration range, where P uptake increases nearly linearly with solution P concentration, the model fitted the data very well, but the uncertainty associated with estimating C_min _for high-P acclimated plants was relatively large. The C_min _values presented for these conditions are therefore uncertain and should be interpreted accordingly.

#### P uptake capacity

**Figure 3:**
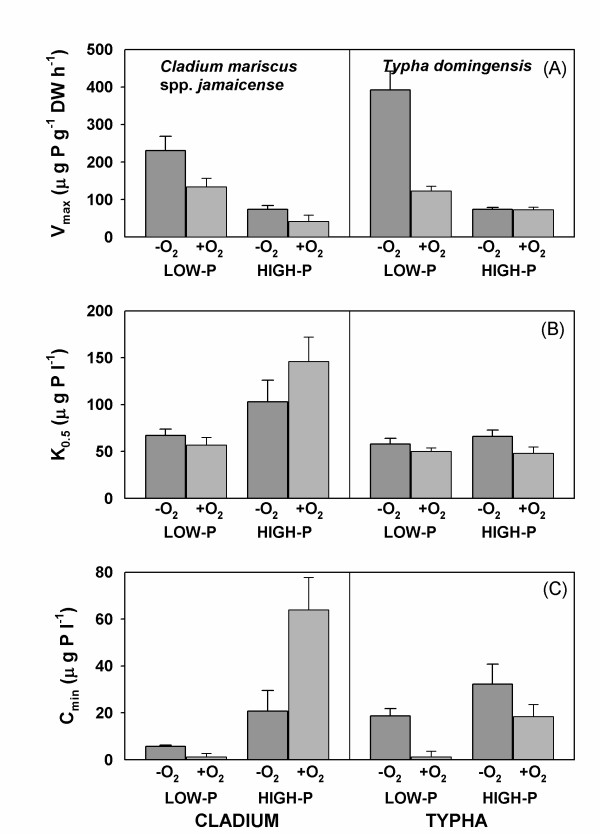
**P uptake kinetic parameters**. Average (± SE) phosphorus (P) uptake kinetic parameters for *Cladium mariscus *spp. *jamaicense *and *Typha domingensis *acclimated to low (5 μg l^-1^) and high (500 μg l^-1^) P concentrations and high (+O_2_: 8 mg l^-1^) and low (-O_2_: <0.5 mg l^-1^) oxygen concentration in the culture solutions. Uptake was measured by P depletions of root solutions followed by stepwise increments of the P concentrations. The kinetic parameters: (A) V_max _(maximum uptake at saturating P concentration), (B) K_0.5 _(half saturation constant), and (C) C_min _(P concentration where there is no net uptake) were estimated by fitting to a modified Michaelis-Menten model.

The saturated rate of P uptake (V_max_) differed significantly between species and was also affected by both P treatment and oxygen level, but the effects of oxygen differed between P treatments as shown by a significant interaction in the ANOVA (table 2). Across treatments the average V_max _was 38% higher in *Typha *than in *Cladium *and the V_max _of low-P acclimated plants was overall more than three-fold higher than the V_max _of high-P acclimated plants (figure 3a and table 2). V_max _was generally more than two-fold higher in the low oxygen treatment compared to the high oxygen treatment, except for *Typha *from the high P treatment, where rates were equal.

**Table 2:** Results of ANOVA for uptake kinetics

Source of variation	V_max_	K_0.5_	C_min_	V_max_/K_0.5_	α
A (species)	5.72*	17.38***	0.07	12.10**	23.05***
B (P level)	76.67***	10.19**	37.66***	56.85***	30.90***
C (O_2 _level)	25.67***	0.53	3.74	8.88**	18.98***
A × B	0.30	9.03**	1.87	0.03	0.01
A × C	0.75	2.62	10.35**	0.17	3.53
B × C	11.50**	0.60	12.03**	6.13*	12.14**

Results of analyses of variance (*F*_(1,33)_-*ratios*) for P uptake kinetic parameters of *Cladium mariscus *spp. *jamaicense *and *Typha domingensis *against species, P level (5 and 500 μg P l^-1^) and oxygen level (8.0 and <0.5 mg O_2 _l^-1^) in the hydroponic culture solution. Significant effects are in bold and indicated by *(P < 0.05), **(P < 0.01) and ***(P < 0.001).

#### Half saturation constant

The half saturation constant (K_0.5_) differed significantly between the two species and was also affected by the P treatment, but the effects of P treatment differed between species as shown by the significant interactions in the ANOVA (table 2). Across treatments the half saturation constants were approximately 70% higher in *Cladium *than in *Typha*, indicating that *Cladium *overall has a lower affinity for P uptake than *Typha *(figure 3b). In *Typha *the half saturation constants did not differ much across treatments, but in *Cladium *the half saturation constants were 1.5-2.5 times higher in the high-P acclimated plants than in low P plants. Oxygen did not significantly affect K_0.5_.

#### Minimum P concentration

The solution P concentration at which there was no net uptake, C_min_, was significantly affected by the treatments as shown by the significant interactions in the ANOVA (table 2). On average, low-P acclimated *Cladium *and *Typha *plants had a C_min _of 3.5 and 9.9 μg P l^-1^, whereas high-P acclimated plants had a C_min _of 43 and 25 μg P l^-1^, respectively (figure 3c). However, in the low P-aerated treatments both species had a low C_min _(1.2 μg P l^-1^). For high-P acclimated plants C_min _was significantly higher (18-64 μg l^-1^) and the effects of oxygen differed between the species (figure 3c).

#### Affinity for P-uptake

The slope of the initial linear part of the uptake curve at low P solution concentrations, α, as well as the ratio between V_max _and K_0.5_, are measures of the uptake affinity. Both affinity measures were significantly affected by plant species, P acclimation and oxygen level, and effects of oxygen level differed between the P treatments (table 2). Overall the affinity for P uptake by *Typha *was two-fold higher than that of *Cladium*, and affinities were 2 to 3 times higher for low-P acclimated plants compared to high-P acclimated plants (table 3). Both species had higher P uptake affinities in low oxygen treatments, and the effects of oxygen were greatest for low-P acclimated plants. The highest affinity was found for low P and low oxygen acclimated *Typha *plants. Numerically, the affinities derived from the initial slope of the curves (α) were higher than the affinity measures derived from the Michaelis-Menten model (V_max_/K_0.5_), but the overall treatment effects were alike.

**Table 3:** Affinity for P uptake

Species	Oxygen	P	V_max_/K_0.5_ (l g^-1^DW h^-1^)	α (l g^-1^DW h^-1^)
*Cladium*	High oxygen	Low	2.43 ± 0.40	0.75 ± 0.17
		High	0.40 ± 0.22	0.28 ± 0.20
	Low oxygen	Low	3.40 ± 0.27	1.44 ± 0.15
		High	0.86 ± 0.20	0.32 ± 0.07

*Typha*	High oxygen	Low	2.51 ± 0.35	0.84 ± 0.14
		High	1.63 ± 0.29	0.90 ± 0.25
	Low oxygen	Low	7.25 ± 1.77	4.08 ± 0.50
		High	1.17 ± 0.13	1.05 ± 0.34

Average (± SE, n = 4-8) ratio between V_max _and K_0.5 _as estimated by the modified Michaelis-menten model (V_max_/K_0.5_) and the slope of the initial part of the uptake curve (α) as a measure of the affinity for P uptake of *Cladium mariscus *spp. *jamaicense *and *Typha domingensis *plants acclimated to low and high P level (5 and 500 μg P l^-1^) and to aerated and low oxygen conditions (8.0 and <0.5 mg O_2 _l^-1^) in hydroponic culture solutions

## Discussion

Ecophysiological studies on nutrient uptake kinetics must be conducted using hydroponically grown plants rather than in soil. Although it is possible to mimic the porewater composition of wetland soils in terms of major nutrient ions and pH, the growth conditions in hydroponic cultures differ significantly from those of wetland soils, particularly oxygen and redox conditions [42-44]. In wetland soils, porewaters are nearly always oxygen-free and may contain variable concentrations of reduced ions and organic compounds resulting in low redox potentials depending on soil organic content, nutrient status and other factors. In hydroponic plant culture, the solution is usually aerated to ensure a good oxygen supply for roots. However, in wetland soils, the root oxygen is delivered from the atmosphere via internal transport through the aerenchyma, and so oxygen supply to support aerobic metabolism within root cells potentially differs considerably [27]. Our experimental conditions mimicked the low oxygen conditions in wetland soils by flushing culture solutions with gaseous N_2_. This treatment maintained oxygen in culture solutions at levels less than 0.5 mg l^-1 ^and so provided a largely anoxic, but not highly reducing, root environment. The growth of the plants was little affected by the oxygen treatments, except for *Typha *root length, which was shorter in the low oxygen treatments.

**Figure 4:**
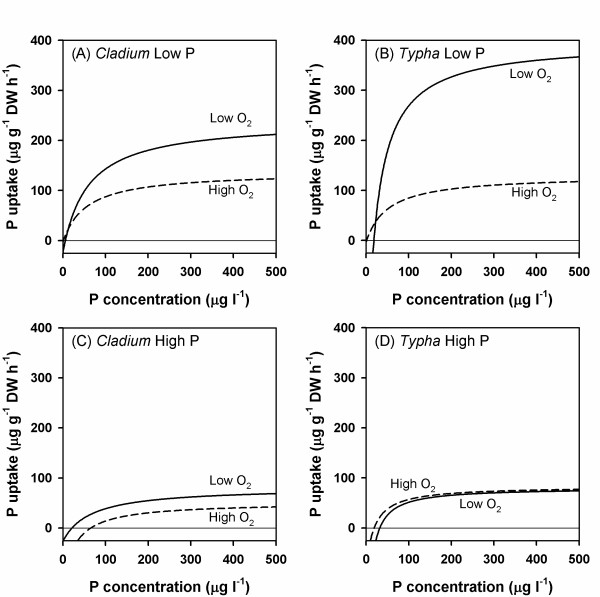
**P uptake curves**. Average estimated uptake of phosphorus (μg P g^-1 ^root dry weight h^-1^) as a function of solution inorganic P concentration for *Cladium mariscus *spp. *jamaicense *and *Typha domingensis *acclimated to low P (5 μg l^-1^) and high P (500 μg l^-1^) concentrations and high oxygen (8 mg l^-1^; dashed lines) and low oxygen (<0.5 mg l^-1^; solid lines) concentration in the culture solutions. The curves were generated by the modified Michaelis-Menten model using the average kinetic parameters for the two species in the different treatments.

Except for *Typha *at high P, tissue P concentrations were higher in the low oxygen treatment (figure 1), and uptake kinetics were also significantly affected by oxygen (figure 4). In waterlogged, anoxic soils, plants have been reported to produce less root biomass with a greater P uptake per unit of root mass than in drained soils [45]. This response has been ascribed to a combination of increased P availability in waterlogged soils and a higher physiological capacity of roots to absorb P, although the mechanisms are not known [45-47]. In the present study, oxygen treatment likely did not affect P availability in the solution cultures. Also, root morphology did not change in response to the oxygen treatments. We, therefore, suggest that the observed differences in uptake characteristics were related to the effects of oxygen on the function and/or expression of the high affinity ion transporters in the root plasma membranes. Plants acquire P in the form of phosphate anions, mostly H_2_PO_4 _^-^, from the soil solution, and uptake occurs against a steep concentration gradient (three orders of magnitude or greater) across the plasmamembrane [34,48]. Many uptake models have been proposed to explain the ability of plants to acquire P both under deficiency and sufficiency conditions [34,35,48,49]. A dual uptake model involving both a high affinity uptake system (HATS) operating at low (<200 μM) concentrations, and a low affinity uptake system (LATS) operating at high (>1 mM) concentrations is widely used to explain the concentration-dependent uptake of nutrient ions [48]. In P-deficient soils only the HATS is operating, apparently with multiple phosphate transporters located in the plasmamembrane through which the energy-mediated uptake occurs [49]. In general, plants respond to nutrient deprivation by increasing uptake affinity as well as uptake capacity [50]. The lack of response to oxygen of high-P acclimated plants, however, indicates that the high affinity transporters are regulated more by P demand than oxygen.

We hypothesized that oxygen-deficient conditions would affect the uptake kinetics of *Cladium *more than that of *Typha *because of differences in their inherent ability to transport oxygen to the roots. This hypothesis could not be verified, because of the confounding effects of P availability. At low P, oxygen affected uptake kinetics more for *Typha *than for *Cladium *whereas at high P the effects were small and mostly on *Cladium*. In earlier studies [[14], and unpublished], low oxygen and particularly reducing (E_h_-150 mV) soil conditions significantly reduced growth and performance of both *Cladium *and *Typha *when P availability was low, but the effects could be largely ameliorated by high P availability. A similar tendency was observed in the present study: effects of oxygen were most pronounced at low P, and nearly disappeared at high P.

Our primary aim was to assess whether differences in P uptake kinetics could explain the observed growth characteristics of the two species in the Everglades. To achieve this goal, we have focused this discussion on plant responses under low oxygen, as this resembles the environmental conditions in the Everglades soils better than the aerated culture solutions. Under low oxygen and high P availability, V_max _did not differ much between the species, but the affinity for P (V_max_/K_0.5 _and α) was significantly lower for *Cladium *than *Typha*. However, *Cladium *had a greater proportion of the biomass allocated to roots, so overall uptake capacity of the two species at high P did not differ much. Root P uptake was largely controlled by the plant P demand. Although we did not investigate ion transport regulation, uptake kinetics may have adjusted via regulation of the membrane-bound high affinity ion transporters [51,52]. These results imply that under P-sufficient conditions, uptake kinetics does not influence competition between the two species. With sufficient P, the plants only need to adjust their uptake system to meet their respective P demands.

At an external concentration of inorganic P of only 5 μg l^-1^, P is certainly growth limiting for both species, as has been shown in many previous studies [14,15]. Plants may respond to such P-deficient conditions in several ways, including allocation of greater mass to roots relative to shoots, and the production of thinner and longer roots enhancing total surface area for nutrient acquisition [53]. In this study, the proportion of biomass allocation to roots increased for both *Cladium *(from 14 to 18%) and *Typha *(from 8 to 15%), but there were no changes in root morphology to increase absorptive area. Both species also adjusted their P uptake system in an attempt to obtain adequate P, but *Typha *more so than *Cladium*. The maximum uptake velocity, V_max_, increased by a factor of 5.3 and 3.1; the half saturation constant, K_0.5_, decreased by a factor 0.14 and 0.54; and the P uptake affinity, V_max_/K_0.5 _and α, increased by a factor 3.9-6.2 and 4.0-4.5 for *Typha *and *Cladium*, respectively. The estimated levels of C_min _(about 6 and 19 μg l^-1 ^in the low-P treatment for *Cladium *and *Typha*, respectively) are obviously too high, as plants were growing and taking up P at 5 μg l^-1^. The depletion methodology used in this study, which included spiking with P to relatively high levels, may have resulted in the build-up of internal pools of P that interfered with the estimation of the true C_min _values. When *Cladium *and *Typha *plants were grown for 30 days in solution cultures with a low supply of P, both species maintained concentrations of 2-3 μg P l^-1 ^in the solutions (unpublished results). We therefore suggest that the true C_min _level for the two species is in this range.

The adjustment of the P uptake system under low P conditions for *Cladium *and *Typha *increased the uptake velocity per unit of root mass 4 to 5-fold compared to the velocities for high-P acclimated plants. Adding the simultaneous increased biomass allocation to roots, the adjustment would result in a 5-fold higher P uptake per plant for *Cladium *and a 10-fold higher uptake for *Typha*. The combined effect of these adjustments is that the P uptake per plant would be alike for low-P acclimated plants at a solution concentration of ~35 μg P l^-1 ^and for high-P acclimated plants at a solution concentration of ~500 μg P l^-1 ^for both species. Actual plant uptake at 5 μg l^-1 ^was of course much lower, as plants grew slower and had lower tissue P concentrations.

Adjustment of the uptake system, particularly V_max_, when plants are exposed to nutrient deficiency is a common response observed by many species and many nutrient ions. The affinity for nutrient uptake as expressed by K_0.5 _is commonly assumed to be less plastic and less affected by plant growth conditions than V_max _[34,45,52]. However, the slope of the uptake curve at low P concentrations, α, and the ratio between V_max _and K_0.5 _(V_max_/K_0.5_) are better measures of affinity than K_0.5_. In the present study, these affinity measures were clearly affected by P availability in a manner similar to V_max_.

A high uptake affinity becomes increasingly important in P-deficient soils, where P uptake is controlled largely by the rate of diffusion to the depleted zones around the roots [54]. The fact that *Typha *adjusts the affinity for P uptake and root biomass more than *Cladium *and that *Typha *has thinner root laterals than *Cladium *[15], increases this species' capacity to extract P from low P solutions relative to *Cladium*. However, despite an apparently less efficient uptake system, *Cladium *outperforms *Typha *during prolonged growth under P-deficient conditions (unpublished). Increased allocation of biomass by *Typha *to roots may reduce its capacity to maintain a balanced acquisition of C and other resources and result in poor growth at P-deficient conditions. Hence, an efficient P uptake system alone does not ensure good performance at persistent low P availability.

Besides optimising the physiology of root P uptake, plants may also increase the availability of P in the rhizosphere by releasing specialized enzymes, known as phosphatases, which hydrolyse soluble organic P derived from soil organic matter to ortho-P for plant uptake [55]. Both *Cladium *and *Typha *secrete phosphatases at low P availability, but the rate of secretion is higher in *Cladium*, enhancing hydrolysis of organic P compounds [56]. Another means of P acquisition from P-deficient soils is symbiosis with arbuscular mycorrhizal fungi [57]. Inoculation of *Cladium *with arbuscular mycorrhizal fungi in a greenhouse pot experiment increased growth and P uptake of *Cladium *significantly [58]. These additional capabilities are clearly important for acquiring sufficient P from the peat-based, low-P soils of the Everglades, where P is stored primarily as organic P with an additional component of Ca-bound P [59,60]. The ability of *Cladium *to access organic P fractions, in concert with its slow growth rate, long tissue life time and high P-use efficiency, likely explains why this species is prolific in the P-deficient Everglades soils.

## Conclusions

A main finding of our study was that *Typha *has a more plastic P uptake system than *Cladium *that allows uptake of P over a wide range of external P concentrations and promotes high growth rates with a relatively low investment in root mass at high P levels. Both species adjust P uptake kinetics in relation to plant demand when P availability is high, but because of its opportunistic traits, *Typha *is more likely to outcompete *Cladium *in P-enriched areas. Under P-deficient conditions *Typha *adjusts P uptake kinetics and biomass allocation to roots more than *Cladium*, and thereby achieves very efficient acquisition of P at low P levels. In contrast, *Cladium *has less need to adjust its P uptake kinetics in response to low P conditions probably because it is already efficient at acquiring P from peat-based soils (e.g., through secretion of phosphatases, symbiosis with arbuscular mycorrhizal fungi, and efficient nutrient conservation growth traits). These findings suggest that differential expression of a similar strategy by *Cladium *and *Typha *under low-P conditions explains why an opportunistic species like *Typha *is able to grow side by side with *Cladium *in the persistently P-deficient Everglades.

## Methods

### Experimental setup

Phosphorus uptake kinetics were measured for whole plants of *Cladium *and *Typha *acclimated in a factorial setup with two levels of P (5 and 500 μg P l^-1^) and two levels of oxygen (8.0 and <0.5 mg O_2 _l^-1^) in hydroponic culture solutions. Since P availability and internal pools of P in the plant tissues are known to affect plant development and physiology, and hence P uptake characteristics, special care was taken to ensure that plants were germinated and propagated at the desired P treatment concentrations. This was achieved by propagating plants from seeds hydroponically in growth cabinets with efficient control of the nutrient composition of the culture solutions. The P uptake kinetic parameters were estimated for whole individual plants of the two species in a controlled environment, the "PhytoNutriTron" (PNT), which is a computer controlled growth facility with four independent steady state hydroponic rhizotrons built into growth cabinets [61].

### Seeds and germination

Seeds of *Typha *and *Cladium *were collected from populations of the two species in the oligotrophic interior of Water Conservation Area 2A in the northern Everglades and germinated on vermiculite at a 14:10 h day:night photoperiod and a 25:10°C thermoperiod, a climatic regime shown to be optimal for germination of the two species [62]. The seedlings were watered with a basic nutrient solution (table 4) at pH 6.5 with additional phosphorus added as K_2_HPO_4 _to obtain the two P levels (5 and 500 μg l^-1^). The composition of the nutrient solution was developed to resemble the porewater concentrations of the major nutrient ions in the interior oligotrophic area of Water Conservation Area 2A of the Everglades. When plants had developed a root system that was large enough to allow the mounting of plants in hydroponic culture (after 3 to 5 months), the seedlings were transferred to a hydroponic nursery culture system.

**Table 4:** Hydroponic nutrient solutions

	Basic nutrient solution	P addition solution	Major adjustment
Condition			
pH	6.5	6.5	NaOH, H_2_SO_4_
Conductivity	1 mS cm^-1^		Intermediate renewal
Temperature	27°C		
Oxygen	<0.5 or 8 mg l^-1^		

Element			
Phosphorus	5 or 500 μg l^-1^	50 mg l^-1^	KH_2_PO_4_
Nitrogen	2.4 mg l^-1^		(NH_4_)_2_SO_4_
Potassium	3.4 mg l^-1^	802 mg l^-1^	K_2_SO_4_, KH_2_SO_4_
Calcium	130 mg l^-1^	291 mg l^-1^	CaSO_4_, CaCl_2_
Sulphur	98 mg l^-1^	690 mg l^-1^	(NH_4_)_2_SO_4_, CaSO_4_, MgSO_4_
Magnesium	41 mg l^-1^	114 mg l^-1^	MgSO_4_
Sodium	50 mg l^-1^	12 mg l^-1^	NaCl
Chloride	216 mg l^-1^	18 mg l^-1^	NaCl, CaCl_2_
Silicium	351 mg l^-1^		Na_2_SiO_3_
Boron	27 μg l^-1^	5.4 mg l^-1^	H_3_BO_3_
Manganese	11 μg l^-1^	2.2 mg l^-1^	MnSO_4_
Zinc	13 μg l^-1^	2.6 mg l^-1^	ZnSO_4_
Copper	13 μg l^-1^	2.6 mg l^-1^	CuSO_4_
Molybdenum	4.8 μg l^-1^	1.0 mg l^-1^	Na_2_MoO_4_
Iron	112 μg l^-1^		FeSO_4_

Composition of the hydroponic nutrient solutions. The basic nutrient solution was developed to resemble the pore water concentration of the major nutrient ions in the interior oligotrophic area of Water Conservation Area 2A of the Everglades. The P addition solution compensated for the uptake of nutrients by plants during growth and was added relative to the depletion of phosphorus by the plants.

### Plant acclimation in nursery system

The plants were acclimated to hydroponic growth at low and high P levels (5 and 500 μg l^-1^) in a nursery system that was set up in a growth chamber operated at a 15 h light/9 h dark cycle, a 30:25°C thermocycle and a 85:90% relative air humidity day:night cycle. Light was provided by a combination of inflorescent light tubes and metal halide bulbs at a photon flux density of 350 μmol m^-2 ^s^-1 ^(PAR) at the base of the plants. Between day and night, the climatic parameters were changed gradually over a one hour transition period. The nursery contained four independent hydroponic growth units, each consisting of one or two 30 litre aerated growth tanks with up to 22 plants. The tanks of each growth unit were connected to a 360 litre external nutrient solution reservoir. The culture solution was recirculated between the reservoir and the growth tanks by pumps delivering 6 litre min^-1 ^of solution to each growth tank. The culture solution consisted of the basic nutrient solution, and phosphorus was adjusted to the experimental levels using the P addition stock solution (table 4). The NH_4 _^+ ^level was adjusted with a solution of (NH_4_)_2_SO_4_. Changes in nutrient concentrations in the culture solutions were minimized through daily monitoring and adjustment of concentration levels. On weekdays, pH was adjusted to pH 6.5, and 112 μg Fe l^-1 ^(FeSO_4_) was added to each unit. Temperature and conductivity were registered and the concentrations of NH_4 _^+ ^and PO_4 _^3- ^were analysed using standard colorimetric methods (Lachat Instruments, Milwaukee, WI, USA). Orthophosphate detection was based on the ascorbic acid method (Method EPA-600/4-79-020, 1983, U.S. Environmental Protection Agency) and NH_4 _^+ ^was analysed using the salicylate method (Ammonia in waters 1981, London, Her Majesty's Stationary Office). Changes in conductivity during operation of the nursery were minimized by intermediate renewal of approximately 75% of the culture solutions when conductivity reached 2 mS cm^-1^. After 2 to 4 months, depending on species and treatment, when the plants started to produce rhizomes and ramets, individual plants were transferred to the controlled environment of the PNT.

### Controlled environment

The uptake experiments were carried out in the PNT, which is a computer controlled hydroponic growth facility for experiments with whole plants [61]. The hydroponic rhizotron system of the PNT consisted of four independent growth units each containing eight root vessels built into a controlled growth chamber in a block design. The growth chamber regulated air temperature, humidity and light intensity (maximum 1200 μmol m^-2 ^s^-1 ^PAR at the base of the plants) in day:night cycles similar to those of the nursery. Each of the four growth units was connected to a separate steady state, temperature (27°C), pH (6.5) and oxygen controlled reservoir (180 l) through which the culture solution was recirculated. The reservoirs were equipped with UV-sterilization units, and the concentrations of NH_4 _^+ ^and PO_4 _^3- ^were monitored continuously by an auto-analyzer using standard colorimetric methods (Lachat Instruments, Milwaukee, WI, USA). The nutrient concentrations were maintained at constant levels through computer-mediated feedback regulation of peristaltic pumps that delivered the P nutrient stock solution and a (NH_4_)_2_SO_4 _stock solution to the reservoirs. The nutrients were supplied continuously at rates equivalent to their depletion in the culture solutions. The reservoirs and the root vessels were sealed from the atmosphere and flushed with either N_2 _gas or atmospheric air to control solution oxygen at the desired levels (<0.5 and 8 mg l^-1^, respectively). Each root vessel (height 700 mm, diameter 80 mm) had a lid with two openings for plants. The culture solution was circulated through each vessel at a rate of 4 litre min^-1^. The use of the P nutrient stock solution and partial replacement of the culture solutions (approximately 60% of the volume) ensured that concentrations of the major nutrients were maintained within ± 10% of desired set point level during the acclimation periods in PNT.

The four growth units of the PNT were used in sequence to create the 8 different treatment combinations (2 P levels × 2 species × 2 O_2 _levels). In order to optimize the control system for P detection, experiments with low and high-P acclimated plants were carried out separately. Between four and eight plants of each species for each treatment were selected at random from the stock of plants in the nursery unit. New ramets, rhizomes and senescent plant parts were removed from the plants before they were mounted in the root vessels of the PNT. The plants were acclimated to the steady state nutrient and oxygen levels in the controlled rhizotrons for at least one month prior to measurement of nutrient uptake.

### Nutrient uptake kinetics

**Figure 5:**
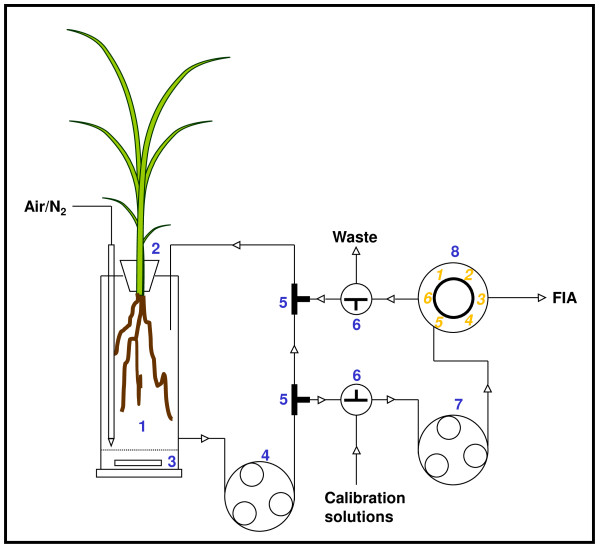
**Experimental chamber used for uptake studies**. Schematic diagram of the experimental system used to estimate the plant P uptake showing the connection between the plant chamber and the flow injection analyzer (FIA) used for PO_4 _^3- ^detection. 1: Acrylic root chamber with diffuser for flushing with air or N_2_; 2: Collar and seal for plant mounting; 3: Magnetic stirring bar protected by a net; 4: High speed peristaltic pump for recirculation of solution in the root chamber; 5: Tee pieces; 6: Three-way valves for calibration of FIA; 7: Peristaltic pump of the FIA; 8: Six port valve of a FIA analyzer channel.

Plant P uptake was measured by the rate of depletion from nutrient solutions using an automated flow injection analyzer with four PO_4 _^3- ^channels (Lachat Instruments, Milwaukee, WI, USA). Orthophosphate detection was based on the ascorbic acid method (Quickchem method no. 10-115-01-1-A (low sensitivity) and 10-115-01-1-B (high sensitivity), Lachat Instruments). At least 16 h before uptake kinetic studies were initiated, four plants were selected at random from the plants in the PNT. The roots were carefully rinsed in nutrient solution, and depending on plant size, transferred to root chambers with a volume between 0.7 and 12 litre (figure 5). The solution levels in the root chambers were marked and the chambers were placed in one of the growth cabinets of the PNT. Each root chamber was placed in a 10 litre tank containing 7 litre of nutrient solution. Centrifugal pumps recirculated water between the tanks and the root chambers at a rate of 1 litre min^-1^. Magnetic stirrers ensured mixing of the nutrient solutions in the root chambers, and depending on treatment, the chambers were flushed with atmospheric air or N_2 _gas at a rate 0.5 litre min^-1^.

One hour before the initiation of the uptake kinetics studies, the circulation between the root chambers and the solution in the external tank was stopped, and the root chambers were flushed five times with a nutrient solution containing 5 μg P l^-1^. Thereafter, uptake studies were initiated by measuring a series of P depletion rates at stepwise increasing levels of P. The concentration of P was increased from 5 to 500 μg P l^-1 ^in steps of 5 to 50 μg P l^-1 ^followed by periods where the rates of depletion were measured. The root chamber was connected to the flow injection analyzer (FIA) through tubing (figure 5). A high-speed peristaltic pump (figure 5, item 4) recirculated solution through two tee-pieces connected to the FIA. The peristaltic pump of the FIA pumped the solution through a three-way valve connected to the recycling flow and to calibration solutions and back into the recycling flow line through the six-port valve of the analyzer. A three-way valve connected the return flow line from the FIA to the drain in order to collect sample water during instrument calibration. The setup ensured that no solution was lost during sampling, except for the volume extracted by the FIA for P analysis. During measurement of P depletion the sample rate was 20 per hour with a sample volume of 780 μl. The extracted volume was replaced with a complete nutrient solution but without P. Calibration of the analyzer channels was performed at regular intervals during the depletion series. The loss of test volume during the measurements due to evapotranspiration, was replaced with distilled water. pH was maintained at 6.5 ± 0.5 by addition of NaOH when necessary.

### Plant tissue measurements

At the end of the depletion experiments, the length of the root and shoot system was measured and plants were separated into roots, leaves, rhizomes and ramets, rinsed in deionised water, and the fresh weights (FW) were recorded before drying in a forced ventilated oven for 48 hours at 80°C for dry weight (DW) determination. The dried plant material was then finely ground and analyzed for N using a N-protein analyzer (Na2000, Carlo Erba, Milan, Italy). The concentrations of P in the plant fractions were analyzed using an ICP-AES (Plasma II, Perkin-Elmer, CT, USA) after HNO_3 _and H_2_O_2 _digestion [63]. The concentration of N and P in the plant fractions and the weight proportions of the fraction were used to calculate the nutrient concentration on a plant basis.

### Estimation of uptake kinetics parameters

The P uptake rates were estimated by linear regression analysis of 3 to 5 consecutive samples of P concentrations measured during depletion. The slopes of the regression lines were corrected for the loss due to sampling by the flow injection analyzer, and divided by root dry weight to obtain the P uptake rate (μg P g^-1 ^root DW h^-1^). Data collected during the initial 8 to 12 minutes after a shift in P concentration in the root chambers were omitted from the analyses in order to ensure that equilibrium between apoplastic and the external P concentrations was achieved.

The relationship between solution P concentration (C) and uptake rates (V) was plotted and fitted by a modified Michaelis-Menten model [38-41]:

The kinetic constants in this model are: V_max_, the maximum uptake velocity at saturating ion concentration; K_0.5 _(= K + C_min_), the half-saturation constant where uptake is 50% of V_max_; and C_min_, the ion concentration at which there is no net inflow. V_max _is a measure of the uptake capacity, and K_0.5 _as well as the ratio between V_max _and K_0.5 _are measures of the affinity of the uptake system. Also the slope, α, of the initial linear part of the uptake curve is a measure of affinity. This model has been successfully used in many studies to assess the underlying mechanisms responsible for up-regulating and down-regulating influx of different nutrient ions into whole plants, as well as the acclimations of the uptake system to different environmental conditions [39,40,61,64-67]. V_max _was entered into the model as a fixed parameter, and was estimated prior to the fitting as the average of the maximum uptake rates registered in a run. The modified Michaelis-Menten equation was fitted to the experimental data by means of a non-linear regression analysis using the Marquardt method (Statgraphics ver. 3, Manugistics, Inc., Maryland, USA). Two measures for P uptake affinity was calculated: (1) the ratio between V_max _and K_0.5 _(V_max_/K_0.5_); and (2) the slope, α, of the initial linear part of the uptake curve as estimated by linear regression analysis.

### Statistics

All statistics were carried out using the software Statgraphics ver. 3 (Manugistics, Inc., Maryland, USA). The difference in uptake kinetic parameters between the two species and the effects of growth P level and oxygen treatment were assessed by a factorial ANOVA model using type III sum of squares. Three-way interactions were ignored in the model. The data were tested for normality and variance homogeneity using Cochran's *C*-test, and data were log-transformed when necessary. Multiple levels of main effects were compared by multiple range tests using the Tukeys HSD procedure at the 5% significance level.

## Authors' contributions

BL carried out the controlled growth experiments and uptake studies and performed most of the statistical analyses. HB and BL drafted the manuscript. IAM, KLM and SLM participated in the design of the study and helped to draft the manuscript. All authors read and approved the final manuscript.

## References

[B1] Davis SM Ogden JC Everglades: the ecosystem and its restoration 1994 Boca Raton, FL: St. Luci Press

[B2] Loveless CM A study of the vegetation in the Florida Everglades Ecology 1959 40 1 9 10.2307/1929916

[B3] McCormick PV Newman S Vilchek LW Landscape responses to wetland eutrophication: Loss of slough habitat in the Florida Everglades, USA Hydrobiologia 2009 621 105 114 10.1007/s10750-008-9635-2

[B4] Vaithiyanathan P Richardson CJ Macrophyte species changes in the Everglades: Examination along a eutrophication gradient J Environ Qual 1999 28 1347 1358

[B5] Rutchey K Vilcheck L Development of an Everglades vegetation map using a spot image and the global positioning system Photogramm Eng Remote Sens 1994 60 767 775

[B6] Rutchey K Schall T Sklar F Development of vegetation maps for assessing Everglades restoration progress Wetlands 2008 28 806 816 10.1672/07-212.1

[B7] Miao S Sindhøj E Edelstein C Allometric relationships of field populations of two clonal species with contrasting life histories, *Cladium jamaicense *and *Typha domingensis* Aquat Bot 2008 88 1 9 10.1016/j.aquabot.2007.08.001

[B8] Miao S Rhizome growth and nutrient resorption: Mechanisms underlying the replacement of two clonal species in Florida Everglades Aquat Bot 2004 78 55 66 10.1016/j.aquabot.2003.09.001

[B9] Miao SL DeBusk WF Reddy KR, O'Conner GA, Schelske CL Effects of phosphorus enrichment on structure and function of sawgrass and cattail communities in the Everglades Phosphorus Biochemistry in Subtropical Ecosystems 1999 New York, Washington DC.: Lewis Publishing 275 299

[B10] Miao SL Sklar FH Biomass and nutrient allocation of sawgrass and cattail along a nutrient gradient in the Florida Everglades Wetlands Ecol Manage 1998 5 245 263 10.1023/A:1008217426392

[B11] Smith SM Leeds JA McCormick PV Garrett PB Darwish M Sawgrass (*Cladium jamaicense*) responses as early indicators of low-level phosphorus enrichment in the Florida Everglades Wetlands Ecol Manage in press

[B12] Ponzio KJ Miller SJ Lee MA Long-term effects of prescribed fire on *Cladium jamaicense *crantz and *Typha domingensis *pers. densities Wetlands Ecol Manage 2004 12 123 133 10.1023/B:WETL.0000021671.65897.0c

[B13] Weisner SEB Miao SL Use of morphological variability in *Cladium jamaicense *and *Typha domingensis *to understand vegetation changes in an Everglades marsh Aquat Bot 2004 78 319 335 10.1016/j.aquabot.2003.11.007

[B14] Lissner J Mendelssohn IA Lorenzen B Brix H McKee KL Miao SL Interactive effects of redox intensity and phosphate availability on growth and nutrient relations of *Cladium jamaicense *(Cyperaceae) Am J Bot 2003 90 736 748 10.3732/ajb.90.5.736 21659170

[B15] Lorenzen B Brix H Mendelssohn IA McKee KL Miao SL Growth, biomass allocation and nutrient use efficiency in *Cladium jamaicense *and *Typha domingensis *as affected by phosphorus and oxygen availability Aquat Bot 2001 70 117 133 10.1016/S0304-3770(01)00155-3

[B16] Newman S Grace JB Koebel JW Effects of nutrients and hydroperiod on *Typha*, *Cladium*, and *Eleocharis*: implications for Everglades restoration Ecol Appl 1996 6 774 783 10.2307/2269482

[B17] Newman S Schuette J Grace JB Rutchey K Fontaine T Reddy KR Pietrucha M Factors influencing cattail abundance in the northern Everglades Aquat Bot 1998 60 265 280 10.1016/S0304-3770(97)00089-2

[B18] Reddy KR DeLaune RD DeBusk WF Koch MS Long-term nutrient accumulation rates in the Everglades Soil Sci Soc Am J 1993 57 1147 1155

[B19] Craft CB Vymazal J Richardson CJ Response of Everglades plant communities to nitrogen and phosphorus additions Wetlands 1995 15 258 271

[B20] Craft CB Richardson CJ Relationships between soil nutrients and plant species composition in Everglades peatlands J Environ Qual 1997 26 224 232

[B21] Richardson CJ King RS Qian SS Vaithiyanathan P Qualls RG Stow CA Estimating ecological thresholds for phosphorus in the Everglades Environ Sci Technol 2007 41 8084 8091 10.1021/es062624w 18186341

[B22] Davis SM Sharitz RR, Gibbons JW Sawgrass and cattail production in relation to nutrient supply in the Everglades 1989 61 U.S. Department of Energy 325 341

[B23] Steward KK Ornes WH Mineral-nutrition of Sawgrass (*Cladium jamaicense *Crantz) in relation to nutrient supply Aquat Bot 1983 16 349 359 10.1016/0304-3770(83)90080-3

[B24] Richardson CJ Ferrell GM Vaithiyanathan P Nutrient effects on stand structure, resorption efficiency, and secondary compounds in Everglades sawgrass Ecology 1999 80 2182 2192 10.1890/0012-9658(1999)080[2182:NEOSSR]2.0.CO;2

[B25] Li SW Lissner J Mendelssohn IA Brix H Lorenzen B McKee KL Miao S Nutrient and growth responses of cattail (*Typha domingensis*) to redox intensity and phosphate availability Ann Bot 2010 105 175 184 10.1093/aob/mcp213 19748907 PMC2794056

[B26] Brix H Sorrell BK Orr PT Internal pressurization and convective gas flow in some emergent freshwater macrophytes Limnol Oceanogr 1992 37 1420 1433

[B27] Sorrell BK Mendelssohn IA McKee KL Woods RA Ecophysiology of wetland plant roots: A modelling comparison of aeration in relation to species distribution Ann Bot 2000 86 675 685 10.1006/anbo.2000.1173

[B28] Bendix M Tornbjerg T Brix H Internal gas transport in *Typha latifolia *L and *Typha angustifolia *L .1. Humidity-induced pressurization and convective throughflow Aquat Bot 1994 49 75 89 10.1016/0304-3770(94)90030-2

[B29] Tornbjerg T Bendix M Brix H Internal gas transport in *Typha latifolia *L and *Typha angustifolia *L .2. Convective throughflow pathways and ecological significance Aquat Bot 1994 49 91 105 10.1016/0304-3770(94)90031-0

[B30] Chabbi A McKee KL Mendelssohn IA Fate of oxygen losses from *Typha domingensis *(Typhaceae) and *Cladium jamaicense *(Cyperaceae) and consequences for root metabolism Am J Bot 2000 87 1081 1090 10.2307/2656644 10947992

[B31] Koch MS Reddy KR Distribution of soil and plant nutrients along a trophic gradient in the Florida Everglades Soil Sci Soc Am J 1992 56 1492 1499

[B32] Davis SM Davis SM, Ogden JC Phosphorus inputs and vegetation sensitivity in the Everglades Everglades. The ecosystem and its restoration 1994 Boca Raton, Florida: St. Lucie Press 357 378

[B33] Palmer ML Mazzotti FJ Structure of everglades alligator holes Wetlands 2004 24 115 122 10.1672/0277-5212(2004)024[0115:SOEAH]2.0.CO;2

[B34] Raghothama KG Phosphate acquisition Annu Rev Plant Phys 1999 50 665 693 10.1146/annurev.arplant.50.1.665 15012223

[B35] Clarkson DT Factors affecting mineral nutrient acquisition by plants Ann Rev Plant Physiol 1985 36 77 115

[B36] Loneraga JF Asher CJ Response of plants to phosphate concentration in solution culture .2. Rate of phosphate absorption and Its relation to growth Soil Sci 1967 103 311

[B37] Keerthisinghe G Hocking PJ Ryan PR Delhaize E Effect of phosphorus supply on the formation and function of proteoid roots of white lupin (*Lupinus albus *L.) Plant Cell Environ 1998 21 467 478 10.1046/j.1365-3040.1998.00300.x

[B38] Barber SA Harley JL, Russell RS Growth requirements for nutrients in relation to demand at the root surface The Soil-Root Interface 1979 London, New York, San Francisco: Academic Press 5 20

[B39] Brix H Dyhr-Jensen K Lorenzen B Root-zone acidity and nitrogen source affects *Typha latifolia *L. growth and uptake kinetics of ammonium and nitrate J Exp Bot 2002 53 2441 2450 10.1093/jxb/erf106 12432036

[B40] Brix H Lorenzen B Morris JT Schierup H-H Sorrell BK Effects of oxygen and nitrate on ammonium uptake kinetics and adenylate pools in *Phalaris arundinacea *L and *Glyceria maxima *(Hartm) Holmb P Roy Soc Edinb B 1994 102 333 342

[B41] Jampeetong A Brix H Effects of NH_4 _+ concentration on growth, morphology and NH_4_+ uptake kinetics of *Salvinia natans* Ecol Eng 2009 35 695 702 10.1016/j.ecoleng.2008.11.006

[B42] Lissner J Mendelssohn IA Anastasiou CJ A method for cultivating plants under controlled redox intensities in hydroponics Aquat Bot 2003 76 93 108 10.1016/S0304-3770(03)00017-2

[B43] Sorrell BK Effect of external oxygen demand on radial oxygen loss by *Juncus *roots titanium citrate solutions Plant Cell Environ 1999 22 1587 1593 10.1046/j.1365-3040.1999.00517.x

[B44] Brix H Sorrell BK Oxygen stress in wetland plants: comparison of de-oxygenated and reducing root environments Funct Ecol 1996 10 521 526 10.2307/2389945

[B45] Rubio G Oesterheld M Alvarez CR Lavado RS Mechanisms for the increase in phosphorus uptake of waterlogged plants: soil phosphorus availability, root morphology and uptake kinetics Oecologia 1997 112 150 155 10.1007/s004420050294 28307564

[B46] Atwell BJ Veerkamp MT Stuiver BCEE Kuiper PJC The uptake of phosphate by *Carex *species from oligotrophic to eutrophic swamp habitats Physiol Plant 1980 49 487 494 10.1111/j.1399-3054.1980.tb03339.x

[B47] Veerkamp MT Corre WJ Atwell BJ Kuiper PJC Growth-rate and phosphate utilization of some *Carex *species from a range of oligotrophic to eutrophic swamp habitats Physiol Plant 1980 50 237 240 10.1111/j.1399-3054.1980.tb04456.x

[B48] Raghothama KG Karthikeyan AS Phosphate acquisition Plant Soil 2005 274 37 49 10.1007/s11104-004-2005-6

[B49] Raghothama KG Phosphate transport and signaling Curr Opin Plant Biol 2000 3 182 187 10837272

[B50] Lee RB Selectivity and kinetics of ion uptake by barley plants following nutrient deficiency Ann Bot 1982 50 429 449

[B51] Jungk A Asher CJ Edwards DG Meyer D Influence of phosphate status on phosphate-uptake kinetics of Maize (*Zea mays*) and Soybean (*Glycine max*) Plant Soil 1990 124 175 182 10.1007/BF00009256

[B52] Drew MC Saker LR Barber SA Jenkins W Changes in the kinetics of phosphate and potassium absorption in nutrient-deficient barley roots measured by a solution-depletion technique Planta 1984 160 490 499 10.1007/BF00411136 24258775

[B53] Fohse D Claassen N Jungk A Phosphorus efficiency of plants .2. Significance of root radius, root hairs and cation-anion balance for phosphorus influx in 7 plant-species Plant Soil 1991 132 261 272

[B54] Jungk A Claassen N Ion diffusion in the soil-root system Adv Agron 1997 61 53 110 full_text

[B55] Jungk A Seeling B Gerke J Mobilization of different phosphate fractions in the rhizosphere Plant Soil 1993 156 91 94 10.1007/BF00024991

[B56] Kuhn NL Mendelssohn IA McKee KL Lorenzen B Brix H Miao SL Root phosphatase activity in *Cladium jamaicense *and *Typha domingensis *grown in everglades soil at ambient and elevated phosphorus levels Wetlands 2002 22 794 800 10.1672/0277-5212(2002)022[0794:RPAICJ]2.0.CO;2

[B57] Rengel Z Mechanistic simulation models of nutrient uptake - a review Plant Soil 1993 152 161 173 10.1007/BF00029086

[B58] Jayachandran K Shetty KG Growth response and phosphorus uptake by arbuscular mycorrhizae of wet prairie sawgrass Aquat Bot 2003 76 281 290 10.1016/S0304-3770(03)00075-5

[B59] Reddy KR Wang Y DeBusk WF Fisher MM Newman S Forms of soil phosphorus in selected hydrologic units of the Florida Everglades Soil Sci Soc Am J 1998 62 1134 1147

[B60] Qualls RG Richardson CJ Forms of soil-phosphorus along a nutrient enrichment gradient in the Northern Everglades Soil Sci 1995 160 183 198 10.1097/00010694-199509000-00004

[B61] Lorenzen B Brix H Schierup H-H Madsen TV Design and performance of the Phyto-Nutri-Tron: a system for controlling the root and shoot environment for whole-plant ecophysiological studies Environ Exp Bot 1998 39 141 157 10.1016/S0098-8472(97)00041-5 11541949

[B62] Lorenzen B Brix H McKee KL Mendelssohn IA Miao S Seed germination of two Everglades species, *Cladium jamaiscense *and *Typha domingensis* Aquat Bot 2000 66 169 180 10.1016/S0304-3770(99)00076-5

[B63] Brix H Lyngby JE Schierup H-H Eelgrass (*Zostera marina *L.) as an indicator organism of trace metals in the Limfjord, Denmark Mar Environ Res 1983 8 165 181 10.1016/0141-1136(83)90049-1

[B64] Jampeetong A Brix H Nitrogen nutrition of *Salvinia natans*: Effects of inorganic nitrogen form on growth, morphology, nitrate reductase activity and uptake kinetics of ammonium and nitrate Aquat Bot 2009 90 67 73 10.1016/j.aquabot.2008.06.005

[B65] Dyhr-Jensen K Brix H Effects of pH on ammonium uptake by *Typha latifolia *L Plant Cell Environ 1996 19 1431 1436 10.1111/j.1365-3040.1996.tb00022.x

[B66] Tylova-Munzarova E Lorenzen B Brix H Votrubova O The effects of NH_4_^+ ^and NO_3_^- ^on growth, resource allocation and nitrogen uptake kinetics of *Phragmites australis *and *Glyceria maxima* Aquat Bot 2005 81 326 342 10.1016/j.aquabot.2005.01.006

[B67] Romero JA Brix H Comín FA Interactive effects of N and P on growth, nutrient allocation and NH_4 _uptake kinetics by *Phragmites australis* Aquat Bot 1999 64 369 380 10.1016/S0304-3770(99)00064-9

